# Emotion Recognition from Large-Scale Video Clips with Cross-Attention and Hybrid Feature Weighting Neural Networks

**DOI:** 10.3390/ijerph20021400

**Published:** 2023-01-12

**Authors:** Siwei Zhou, Xuemei Wu, Fan Jiang, Qionghao Huang, Changqin Huang

**Affiliations:** Key Laboratory of Intelligent Education Technology and Application of Zhejiang Province, Zhejiang Normal University, Jinhua 321004, China

**Keywords:** emotion recognition, large-scale video clips, deep convolutional neural network, attention mechanism, cross-channel, deep feature fusion

## Abstract

The emotion of humans is an important indicator or reflection of their mental states, e.g., satisfaction or stress, and recognizing or detecting emotion from different media is essential to perform sequence analysis or for certain applications, e.g., mental health assessments, job stress level estimation, and tourist satisfaction assessments. Emotion recognition based on computer vision techniques, as an important method of detecting emotion from visual media (e.g., images or videos) of human behaviors with the use of plentiful emotional cues, has been extensively investigated because of its significant applications. However, most existing models neglect inter-feature interaction and use simple concatenation for feature fusion, failing to capture the crucial complementary gains between face and context information in video clips, which is significant in addressing the problems of emotion confusion and emotion misunderstanding. Accordingly, in this paper, to fully exploit the complementary information between face and context features, we present a novel cross-attention and hybrid feature weighting network to achieve accurate emotion recognition from large-scale video clips, and the proposed model consists of a dual-branch encoding (DBE) network, a hierarchical-attention encoding (HAE) network, and a deep fusion (DF) block. Specifically, the face and context encoding blocks in the DBE network generate the respective shallow features. After this, the HAE network uses the cross-attention (CA) block to investigate and capture the complementarity between facial expression features and their contexts via a cross-channel attention operation. The element recalibration (ER) block is introduced to revise the feature map of each channel by embedding global information. Moreover, the adaptive-attention (AA) block in the HAE network is developed to infer the optimal feature fusion weights and obtain the adaptive emotion features via a hybrid feature weighting operation. Finally, the DF block integrates these adaptive emotion features to predict an individual emotional state. Extensive experimental results of the CAER-S dataset demonstrate the effectiveness of our method, exhibiting its potential in the analysis of tourist reviews with video clips, estimation of job stress levels with visual emotional evidence, or assessments of mental healthiness with visual media.

## 1. Introduction

The emotion of humans is an important indicator or reflection of their mental states, e.g., satisfaction or stress, and recognizing or detecting emotion from different media is essential to perform sequence analysis or different applications [1,2], e.g., mental health assessments, job stress level estimation, and tourist satisfaction assessments. As visual media (e.g., images or videos) of human behaviors contain plentiful emotional cues, the automatic recognition of human emotions from these visual materials, as a trending research field of computer vision, has received much attention in the past decade due to its significance in a wide range of computer vision applications, such as education [3], human–computer interaction [4,5], behavioral science [6], security [7] and health care  [8,9,10]. As an important basis for emotion science research, emotion category classification models mainly include discrete classification representation and continuous dimension representation [11]. The discrete emotion classification model universally defines emotional states with categories in discrete feature space, such as the seven basic emotions: angry, disgust, fear, happy, neutral, sad, and surprised. In this paper, we focus on developing a robust deep-neural-network-based technique to perceive the discrete emotional state of the subject from large-scale videos or images, and we lay a solid foundation for their application, e.g., in the analysis of tourist reviews with video clips, the estimation of job stress levels with visual emotional evidence and the assessment of mental health with visual media.

**Figure 1:**
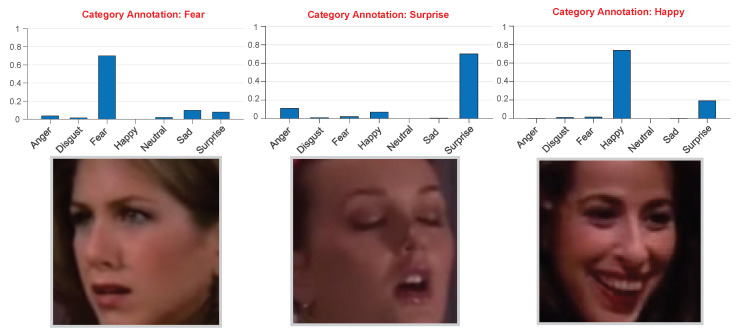
Human facial expressions, in reality, can be regarded as a combination of basic expressions. The label distributions on the facial images are the output of the ResNet-50 network trained on facial regions of the CAER-S dataset.

Researchers have recently introduced different data modalities from traditional emotion recognition tasks, such as visual, speech, and text features [12,13,14,15], to conduct more specific research on human emotion perception. Facial expression has been the de-facto standard for emotion recognition research among the various data modalities. It is considered the most effective non-verbal emotion medium, containing the most abundant, salient, and directly explicit emotional information. Emotion recognition based on facial expressions has also attracted much research attention. In recent years, with the support of backbone networks, such as convolutional neural network (CNN) and vision transformer (ViT), facial expression recognition (FER) methods based on deep neural networks have achieved impressive performance improvements on major benchmarks [16,17]. However, many previous studies have revealed that emotion recognition methods that only consider facial information in real applications often fail to produce stable and ideal results. This is mainly because (1) a specific facial expression itself in the wild is a combination of basic emotions, as shown in Figure 1, which can be difficult and confusing to identify and (2) the vital role of context information, such as gestures, interpersonal interactions, and scenes, which can compensate for limited emotion information, is ignored. Recently, many researchers focusing on context-aware emotion recognition have revealed that such context information, which is regularly utilized as a reference for emotion evaluation, affects and indicates the emotional state of the subject visually. Therefore, with the additional consideration of context information, methods for context-aware emotion recognition have been investigated to alleviate the limitations of the aforementioned FER-based emotion recognition methods.

**Figure 2:**
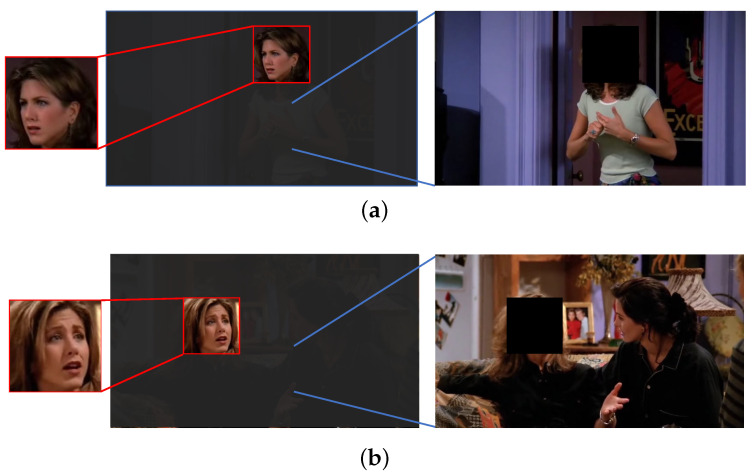
Intuition of emotion understanding: for the images in (**a**,**b**), the complementary information between facial expressions and context can rectify our understanding of their respective implied emotion information, which enables us to perceive the emotional states more precisely.

**Figure 3:**
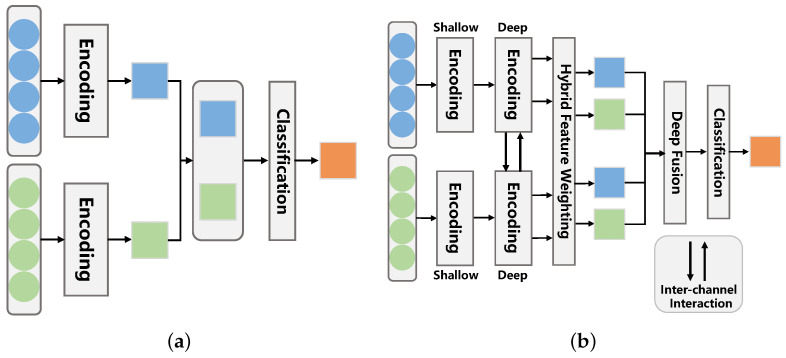
Intuition of CAHFW-Net: For images as in (**a**,**b**), conventional CAER methods that fuse the features using single-level simple concatenation only as in (**a**) often fail to overcome emotion confusion. Unlike these methods, CAHFW-Net introduces inter-channel interaction to capture complementary information between face and context as in (**b**).

While the additional analysis of context information provides us with information compensation related to emotion perception, the efficient extraction and utilization of complementary information between face and context features cannot be underestimated, which is helpful in understanding the emotion cues underlying respective features. Given cropped face images, as shown in Figure 2a,b, it is difficult for us to identify the emotional states of the subjects only by virtue of facial cues, such as muscle curves (from their facial expressions it seems the subjects are feeling either sad or surprise (in Figure 2a) and surprise or fear (in Figure 2b)). However, when we present the context information, such as body movements and the context in which they are, as shown in Figure 1, we can more readily guess their emotional states as surprise and sad, respectively, based on the combination of facial expressions and context information. The above emotion information processing, which can be summarized as “perceiving the emotional state expressed by the subject under the premise of understanding where and what the subject is doing”, implicitly contains the perception of inter-feature complementary gains. Focusing on the information interaction and complementarity between face and context can enable us to rectify the emotion information understanding of respective features, thereby obtaining the emotion cues most related to emotion recognition. However, neither the approaches from the coarse-grained perspective of face-context dual-channel [18,19] nor the approaches from the multi-channel fine-grained perspective with consideration of face, pose, and non-subject information [20,21] take into account the effective information interaction between feature streams, as shown in Figure 3a.

To address these issues, in this paper, we propose a novel emotion evaluation framework named the cross-attention and hybrid feature weighting network (CAHFW-Net) to more precisely evaluate human emotions from images from large-scale video clips containing context information with human facial expression and context information in a joint, interactive and complementary manner (as shown in Figure 3b). We focus on extracting and utilizing the complementary information between the features of the face and context, instead of simple feature fusion using direct concatenation like most existing context-aware emotion recognition (CAER) methods. Our approach has three stages: shallow encoding, deep encoding, and deep fusion. First, in the shallow encoding stage, a CNN-based dual-branch encoding (DBE) architecture is designed to extract the shallow features of facial expressions and context simultaneously. Second, in the deep feature encoding stage, we propose a hierarchical-attention encoding (HAE) network to obtain the adaptive emotion features, which will be fused to predict the final individual emotional state. Specifically, we define an interaction–rectification (I-R) pair, in which a cross-attention (CA) block is designed to generate informative complementary features by considering the correlation and heterogeneity between the features of both face and context in a cross-channel manner. Then, an element recalibration (ER) block is introduced behind the CA block to revise each channel’s feature map by embedding global information, thereby capturing the salient emotion cues and compressing the worthless information. Furthermore, an adaptive-attention (AA) block is designed to obtain a set of optimal weighted features with its core operation, namely hybrid feature weighting. Eventually, in the deep fusion stage, the emotion-adaptive features in the above-mentioned feature set will be fused via a deep fusion (DF) block hierarchically and densely to predict the final emotion category.

The main contributions of this paper can be summarized as follows:

- We propose an interaction–rectification pair constructed with cross-attention and element recalibration blocks in the deep encoding stage of the network (as shown in Figure 3b). This module adopts the CA block to capture complementary information between facial expression and context features. Moreover, the introduced ER block can further rectify the model’s emotion understanding by embedding global information into respective feature maps.
- To effectively integrate the features of face and context channels, we develop the AA block to obtain the optimal weighted features via a strategy named hybrid feature weighting. Additionally, a DF block is defined to fuse these features hierarchically and densely for final classification (as presented in Figure 3b).
- We use the proposed DBE network, I-R pair module, AA, and DF blocks to construct a novel deep architecture, i.e., the cross-attention and hybrid feature weighting network (CAHFW-Net), to predict individual emotional states. Experiments are performed on a publicly available dataset (i.e., the CAER-S emotion dataset) to demonstrate the contribution of each block and confirm the effectiveness of our method.

The remainder of this paper is organized as follows. Section 2 overviews the work related to context-based emotion recognition methods and the attention mechanism in deep neural networks. Section 3 provides a detailed description of the proposed framework and algorithm. Our experimental steps and results are presented in Section 4. Finally, the conclusions are provided in Section 5. To help readers understand our work better, some notations in this paper are summarized in Table 1.

## 2. Related Work

Our work relates to two major research directions: context-aware emotion recognition (CAER) and the attention mechanism in deep neural networks. Here, we focus on several representative methods closely related to our work.

### 2.1. Context-Aware Emotion Recognition

Since the human face contains strong salient information that is conducive to extracting more refined emotion information, such as micro-expressions [22,23,24], the research on human emotion recognition methods throughout the past decade has focused on facial expression analysis [25,26,27,28,29]. Traditional research either uses facial fiducial points based on the Gabor-feature facial point detector [30] or focuses on facial action unit detection where a set of facial muscle movements is utilized for encoding corresponding facial expressions [31,32]. Compared with traditional methods, deep neural networks, such as deep convolution neural networks (CNNs) and vision transformer (ViTs)-based networks, can extract deeper and more contextual information [33,34]. To capture the temporal dynamic variations of expression intensities among consecutive video frames, several techniques have also been introduced to make interactions across the time axis utilizing 3D-CNNs or graph convolution networks combined with Bi-LSTM [35,36,37]. However, the aforementioned methods that primarily rely on face analysis fail to take into account the context information in sample mining, which means the model cannot overcome the problems of emotion confusion and misunderstanding (as described in Section 1). Furthermore, in terms of practical applications, faces’ occlusion and uneven surface illumination limit models’ steady and efficient performance. This impedes the process of expanding related research to real-world settings.

Psychological research [38,39,40] has shown that similar to most psychological processes, emotion processes cannot be interpreted in isolation without context information. To address the limitations of limited emotion information on facial regions on emotion recognition tasks, several methods integrating visual cues such as body and scene context into encoding streams have been proposed [18,19,20,21], resulting in the evolution of emotion recognition methods from facial feature analysis to context-aware emotion recognition. Kosti et al. [18] and Lee et al. [19] make significant strides in the CAER task, proposing a similar two-stream feature extraction architecture considering the object’s body (face for [19]) and context features jointly. Lee et al. [19] propose the CAER dataset and leverage the attention mechanism into the CAER task for the first time; therefore, the model can better examine the importance of face and context features. From a fine-grained perspective, Mittal et al. [20] fuse face, pose, context, socio-dynamic context, and inter-agent interactions to jointly analyze the emotional state. Thuseethan et al. [21] additionally consider facial expressions and pose information of non-target subjects in the same context. At the same time, a novel hybrid feature fusion method is proposed to obtain fine-grained information from feature interaction.

However, most of the aforementioned methods, which still merely adopt single-level simple concatenation for feature fusion (as presented in Figure 3a), fail to effectively capture the interactive information and weaken the complementary gains among feature streams.

### 2.2. Attention Mechanism in Deep Neural Networks

Interest in the attention mechanism, which enables humans to capture valuable goal-oriented information in complex situations, has recently become a hot topic in research. The past few years have witnessed various approaches for visual tasks attempting to introduce various attention mechanisms into deep neural networks (e.g., CNN, ViT) with success. For the unimodal encoding framework, recent studies mainly focus on the attention operation on a homogeneous feature map. Hu et al. [41] investigated the channel relationship between network features and proposed a squeeze-and-excitation block, which highlights the beneficial features and suppresses the less useful ones to retain the most valuable channel information. To emphasize the salient and meaningful features along the channel and spatial axes, Woo et al. [42] proposed the convolutional block attention module (CBAM), which sequentially combines two sub-modules (i.e., channel and spatial attention modules) so that each of the branches can learn “what” and “where” to allocate attention in the above two principal dimensions, respectively. Wu et al. [43] propose a collaborative multi-attention module to extract the collaborative information of the corresponding foreground object by using self-attention to the activation maps of multi-images in the task of weakly supervised semantic segmentation. Furthermore, Wang et al. [44] propose a self-supervised equivariant attention mechanism to discover additional supervision and narrow the gap between fully and weakly supervised semantic segmentation, and improve the network ability for consistent prediction by incorporating self-attention with equivariant regularization. To improve the performance of face forgery detection for images with low quality and/or diverse sources, Lin et al. [45] propose an improved Xception method by embedding the dual-attention feature (i.e., the CBAM) into the original Xception model, which enables the network to refine and reorganize the high-semantic features captured by the middle flow of Xecption. For multimodal features, the extension of attention modules aims to capture the interactive information among features in multi-stream architecture. Kim et al. [46] proposed a bilinear attention network to exploit bilinear interactions between input channels of two different modalities. Meanwhile, the joint representations for each pair of channels are extracted by using low-rank bilinear pooling. Nagrani et al. [47] proposed a novel ViT-based architecture named multimodal bottleneck transformer, which restricts the stream of cross-modal information among latent units to condense the most related inputs in each modality through tight fusion bottlenecks. Chen et al. [48] migrated multi-scale feature representation learning from CNN [49,50] to ViT and proposed the CA mechanism to exchange information with non-patch tokens from two feature streams with different patch sizes. Zhou et al. [51] leverage the motion cues implied in optical flow features as a cross-channel and bottom-up signal to guide the model’s perception of object appearance in input images by using the proposed motion-attentive transition module, which is constructed with the soft attention unit and attention transition unit.

Along the same line of exploring the complementary effect between the features of different views, as shown in the network constructed by Zhou et al. [51], we adopt a cross-channel operation into our network to fully capture and exploit the complementary information between the face and context features. However, compared with the cross-channel operation in [51] (i.e., the motion-attentive transition), our method (i.e., the CA block) can be regarded as an extension of self-attention from unimodal feature processing to multimodal processing, which is task-specific and has a distinctly different computational graph.

## 3. CAHFW-Net Framework for Context-Aware Emotion Recognition

**Figure 4:**
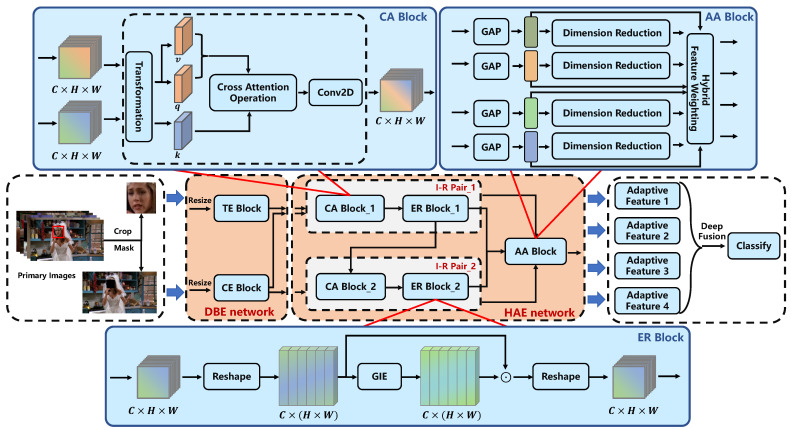
The proposed cross-attention and hybrid feature weighting network (CAHFW-Net) is used for automatic emotion prediction. The dual-branch encoding (DBE) network is constructed with target encoding (TE) (dealing with facial regions) and context encoding (CE) blocks, by which a pair of face-context images can be encoded into respective shallow representations. *C*, *H* and *W* denote the number of channels, the height, and width of the input tensor, respectively. ⊙ refers to element-wise multiplication. I-R denotes the interaction–rectification pair constructed with cross-attention (CA) and element recalibration (ER) blocks. The adaptive-attention (AA) block is used to find the optimal fusion weights for feature integration. GIE refers to Global Information Extraction. GAP and Conv2D denote the global average pooling and two-dimensional convolution layer. The loss function for the CAHFW-Net is cross-entropy (CE), as shown in Equation (27).

This paper uses the proposed CAHWF-Net to evaluate individual emotional states by considering the complementary gains implied in the correlation and heterogeneity between face and context features. Specifically, the face and context images are denoted as $I_{F}=\left\{I_{F}^{1},\cdots ,I_{F}^{N}\right\}$ and $I_{C}=\left\{I_{C}^{1},\cdots ,I_{C}^{N}\right\}$, respectively, where *N* is the number of images. Our ultimate objective is to infer the emotional states *p* among *K* emotion labels $\left\{y_{1},\cdots ,y_{K}\right\}$ on discrete space. Our model first obtains the shallow representation pair of face and context images via the DBE network, constructed with TE and CE blocks. Second, the complementary information is mined and embedded into corresponding feature maps through the CA block and ER block in the I-R pair of the HAE network, respectively. Finally, the AA block in the HAE network produces adaptive emotion features, which then serve as the inputs of the DF block to estimate the current emotional state. Figure 4 illustrates the overall pipeline of our proposed framework. In the following, we describe the DBE network, CA block, ER block, AA block and DF block according to the above model flow.

### 3.1. Representation Generation by Dual-Branch Encoding Network

The experimental results presented in [52,53] illustrate the strong representation capacity and promising performance of CNN-based models. Hence, to represent facial and contextual information, with a proper account of the trade-off between performance and parameters, we develop a lightweight DBE network to extract the facial and contextual feature maps, which includes TE and CE blocks, as shown in Figure 4. We first detect and crop the facial regions from the original images using the CNN-based face detectors available in the off-the-shelf library, namely Dlib [54], to build the input set $I_{F}$ to feed into the TE block. Secondly, to locate the semantic components containing more discriminative emotion cues, a masking mechanism is introduced to build the input set $I_{C}$ for the CE block. The *i*-th masked contextual image, $I_{C}^{i}\in R^{224\times 224}$, for an input image $I_{i}$ is given as Equation (1).

(1) $$I_{C}^{i}=\left\{\begin{array}{cc} I_{i}\left(x,y\right) & ifI_{i}\left(x,y\right)\notin {bbox}_{face},\;i\in N,\\ 0 & otherwise, \end{array}\right.$$

where $bbox_{face}$ denotes the bounding box with the coordinate (x,y) produced from face detector.

**Figure 5:**
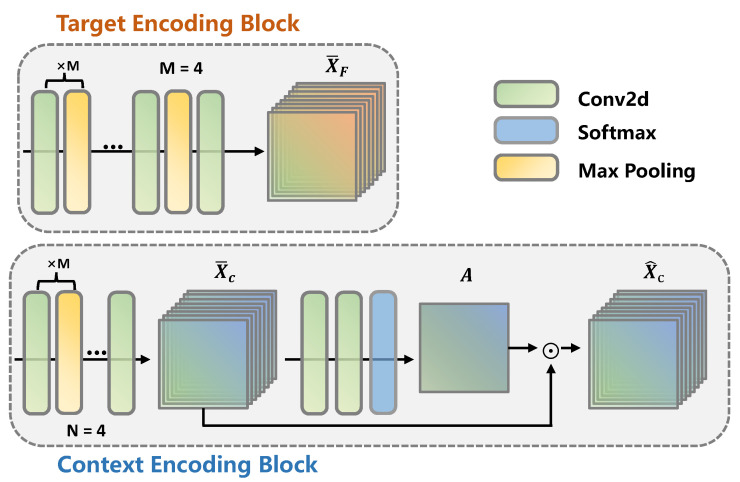
The shallow feature maps of the face and context branches are extracted via a parallel encoding process using the face and context encoding blocks. ${\bar{X}}_{F}\in R^{C\times H\times W}$ and ${\hat{X}}_{C}\in R^{C\times H\times W}$ are the corresponding results. ⊙ denotes the element-wise multiplication. “×M” means that the enclosed part is performed M times.

The dual-branch encoding method achieves representation generation with its core blocks, namely the TE and the CE blocks. As shown in Figure 5, the TE block and the front part of the CE block are built with five two-dimensional convolution blocks in the same stacking manner. Mathematically, the former four 2D convolution blocks and the 5-th one can be expressed as Equations (2) and (3), respectively.

(2) $$F_{t}^{k+1}=M\left(\delta \left(B\left(C_{2}\left(X_{t}^{k}\right)\right)\right)\right),\;k=0,1,2,3,$$

(3) $$F_{t}^{5}=\delta \left(B\left(C_{2}\left(X_{t}^{4}\right)\right)\right),\;t\in \left\{F,\;C\right\},$$

where *t* is the type of input tensors, and F and C refer to face and context, respectively. $X_{t}^{k}$ is the (k+1)-th input tensor of $C_{2}$ layer in face and context encoding blocks. $C_{2}$ is a two-dimensional convolution (i.e., a Conv2D layer), while B(.), δ(.) and M(.) refer to the batch normalization, ReLU, and max-pooling functions. The whole feedforward process described above can be expressed as

(4) $${\bar{X}}_{t}=F_{t}\left(X_{t},\;W_{t}\right)\in R^{C\times H\times W},$$

where $W_{t}$ is the parameters for the encoding layers. $F_{t}\left(.\right)$ denotes the stacked two-dimensional convolution blocks constructed, as shown in Equation (5), while ${\bar{X}}_{t}\in \left\{{\bar{X}}_{F},\;{\bar{X}}_{C}\right\}$ is the representation generated via the above process and C×H×W is the shape of ${\bar{X}}_{t}$.

(5) $$F_{t}=\left[F_{t}^{1},F_{t}^{2},\cdots ,F_{t}^{5}\right].$$

Additionally, an attention-based highlight module is developed and appended at the end of the CE block (as shown in Figure 5), which takes the intermediate feature map ${\bar{X}}_{C}\in R^{C\times H\times W}$ as the input to the inference of an attention map $A\in R^{H\times W}$, to further enable the CE block to locate the salient context regions and extract discriminative emotion cues. H×W is the spatial resolution of each channel map of ${\bar{X}}_{C}$. The complete process of the attention-based highlight module can be expressed as shown in Equations (6) and (7).

(6) $$A=\sigma (F_{AH}^{2}\left(F_{AH}^{1}\left({\bar{X}}_{C}\right)\right)\in R^{H\times W},$$

(7) $${\hat{X}}_{C}=A⊙{\bar{X}}_{C},$$

where σ refers to the softmax function, and $F_{AH}$ is the two-dimensional convolution layers in the attention-based highlight module as expressed in Equation (8)

(8) $$F_{\mathrm{AH}}^{i}=\delta \left(B\left(C_{2}\left({\bar{X}}_{C}\right)\right)\right),\;i=1,2.$$

As described above, the dual-branch encoding network utilizes a lightweight CNN-based framework to produce shallow representations of the face and context branch in parallel. Note that the attention map, serving the model to extract discriminative emotion cues, is implicitly learned in an unsupervised manner.

### 3.2. Hierarchical Cross-Attention Block and Element-Recalibration Block for Feature Interaction and Rectification

The cross-channel attention mechanism, with its strong ability to capture inter-feature correlation and heterogeneity, has certain advantages when it comes to processing the multi-modality and the multi-view data in the fields of emotion recognition [47], object detection [55] and image classification [48]. Hence, in this paper, we propose a novel hierarchical cross-attention method to extract the complementary information between face and context features in a cross-channel manner, which benefits the model’s understanding of emotion cues and emotional state prediction.

The aforementioned inter-channel interaction process is conducted through two tiers of the I-R pair, as shown in Figure 4. Each I-R pair is constructed with one CA block and one ER block, where the CA block extracts the inter-feature complementary information and the ER block embeds the global information to the respective representation, to recalibrate the model’s emotion understanding of the feature regions by sufficiently utilizing the complementary information previously obtained.

**Figure 6:**
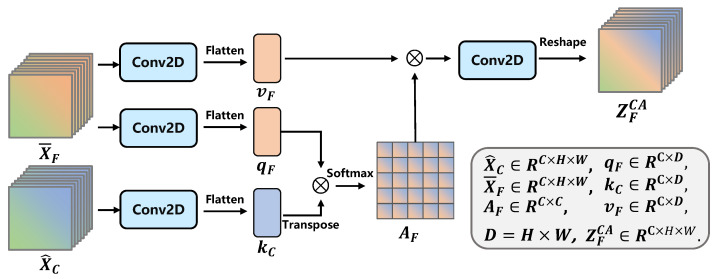
Architecture of cross-attention in the fist I-R pair. The complementary information is extracted via a cross-channel interaction using the CA operation. $Z_{F}^{\mathrm{CA}}\in R^{C\times H\times W}$ denotes the corresponding output. The detailed shape of each tensor is presented in this figure.

In more detail, for the given facial feature map ${\bar{X}}_{F}\in R^{C\times H\times W}$ and contextual feature map ${\hat{X}}_{C}\in R^{C\times H\times W}$ produced, respectively, via the TE block and CE block, the CA operation of the first tier of the inter-channel interaction (as shown in Figure 6) can be expressed using Equations (9) and (10).

(9) $$Y_{F}^{\mathrm{CA}}=\mathrm{Softmax}\left(\frac{Q_{F}K_{C}^{⊤}}{\sqrt{D}}\right)V_{F}\in R^{C\times D},$$

(10) $$Z_{F}^{\mathrm{CA}}=\delta (B(C_{2}\left(Reshape\left(Y_{F}^{\mathrm{CA}}\right)\right)\in R^{C\times H\times W}$$

where T refers to matrix transposition. The query, key, and value of the face and context features can be obtained using Equation (11), while *D* is the product of *H* and *W*.

(11) $$\left\{\begin{array}{c} Q_{F}=F_{Q}\left({\bar{X}}_{F}\right)\in R^{C\times D}\\ K_{C}=F_{K}\left({\hat{X}}_{C}\right)\in R^{C\times D}\\ V_{F}=F_{V}\left({\bar{X}}_{F}\right)\in R^{C\times D} \end{array}\right.,$$

In our method, for feature map $X=\left\{{\bar{X}}_{F},{\hat{X}}_{C}\right\}\in R^{C\times H\times W}$, we adopt Conv2D layers to produce the raw query, key, and value. To obtain the $Q_{i}$, $K_{i}$ and $V_{i}$ in Equation (x), we flatten the respective query, key, and value at the last dimension of the tensor. Thus, $F_{Q}\left(.\right)$, $F_{K}\left(.\right)$ and $F_{V}\left(.\right)$ can uniformly be expressed as Equation (12).

(12) $$F_{Q,K,V}=F_{flatten}\left(C_{2}\left(X\right)\right).$$

where X is the feature map from the TE and CE blocks, i.e., ${\bar{X}}_{F}\in R^{C\times H\times W}$ and ${\hat{X}}_{C}\in R^{C\times H\times W}$.

The complementary information is then extracted via Equation (9) and via Equation (10); the output is denoted as $Z_{F}^{\mathrm{CA}}$. Figure 6 illustrates the process of capturing inter-feature complementary information through cross-attention. We can treat the above process as a preliminary rectification to the model’s understanding of the facial feature.

Recently, the successful introduction of the ER block [56] and the squeeze-and-excitation block [57] has resulted in the significant improvement of the model’s representation ability brought using global information embedding. Thus, to further boost the network’s emotion representation ability, an ER block (as shown in Figure 4) is introduced to utilize the underlying complementary information by embedding the global information into the whole feature map. The generation of global information $G_{F}$ (i.e., GIE in Figure 4) and recalibration for the feature element can be expressed as shown in Equations (13) and (14), respectively.

(13) $$G_{F}=\mathrm{Softmax}\left(\bar{Z_{F}}W_{F}^{\mathrm{TM}}{\bar{Z_{F}}}^{⊤}\right)⊗\bar{Z_{F}}\in R^{C\times (H\times W)},$$

(14) $$R_{F}^{\mathrm{ER}}=\mathrm{Reshape}\left(\bar{Z_{F}}⊙G_{F}\right)\in R^{C\times H\times W},$$

where $\bar{Z_{F}}\in R^{C\times (H\times W)}$ is the matrix constructed by flattening the $Z_{F}^{\mathrm{CA}}\in R^{C\times H\times W}$ at its height dimension, while $W_{F}^{\mathrm{TM}}$ is the transformation matrix, i.e., the weight of a Conv1D layer. ⊗ and ⊙ represent matrix multiplication and element-wise multiplication, respectively.

The process of the second I-R tier to obtain the rectified feature $R_{C}^{\mathrm{ER}}$ of the context branch is similar to that described above, except for the generation of the query, key, and value for the CA operation. The query, key, and value of the features of the two channels can be obtained using Equation (15).

(15) $$\left\{\begin{array}{c} Q_{C}=F_{Q}\left({\hat{X}}_{C}\right)\in R^{C\times D}\\ K_{Z_{F}}=F_{K}\left(R_{F}^{\mathrm{ER}}\right)\in R^{C\times D}\\ V_{C}=F_{V}\left({\hat{X}}_{C}\right)\in R^{C\times D} \end{array}\right.\;\;\;\;,$$

From the above process, it can be observed that CA takes the feature maps of the face and context channel as a multi-view objective and processes the inter-channel representations interactively. Therefore, the cross-attention operation can seamlessly mine the complementary information by considering inter-channel feature interaction, which can significantly improve the model’s understanding of emotion cues hidden in the individual and context regions of the images. Furthermore, from the pipeline of the introduced ER block, we can see that a Gram-like matrix, which implies the element correlation between $\bar{Z_{F}}$ and $W_{F}^{\mathrm{TM}}{\bar{Z_{F}}}^{⊤}$, is obtained using Equation (13) without softmax for normalization. Such a matrix reveals the trade-off among feature elements, that is, the greater the original value in the feature maps of $\bar{Z_{F}}$ and $W_{F}^{\mathrm{TM}}{\bar{Z_{F}}}^{⊤}$, the greater the value in the same dimension of the Gram-like matrix. In other words, in line with the principle that the larger the eigenvalue, the more important the element, the ER block can highlight the feature elements that are beneficial for emotional state prediction and suppress the less valuable ones. Hence, the ER block has advantages in extracting the more salient representation of emotion cues.

### 3.3. Adaptive-Attention Block and Deep Fusion Block to Combine Features

To recognize the final emotional state by combining the face and context effectively, the approach in [19] uses an adaptive fusion network which combines the facial features and contextual features by using a feature weighting operation, which can be expressed as in Equations (16) and (17). The above operation infers the optimal fusion weights for the respective features similarly using an attention module to Equation (6) to alleviate the limitations of the previous methods where a direct concatenation of varied features fails to achieve subtle and optimal performance.

(16) $$\lambda =\sigma \left(\prod \left(C_{1}\left(C_{1}\left({\tilde{X}}_{F}\right)\right),C_{1}\left(C_{1}\left({\tilde{X}}_{C}\right)\right)\right)\right)\in R^{1\times 2}$$

(17) $$X_{fusion}=\prod \left({\tilde{X}}_{F}⊙\lambda_{F},{\tilde{X}}_{C}⊙\lambda_{C}\right)\in R^{\left(2\times C\right)}$$

where ∏(.) is a concatenation operator. ${\tilde{X}}_{F}$ and ${\tilde{X}}_{C}$ are the tensors generated by the GAP layers with the feature maps from the CE and TE blocks as inputs. $\lambda =[\left[\lambda_{F},\;\lambda_{C}\right]]$ refers to the fusion weight list, and σ refers to softmax function.

**Figure 7:**
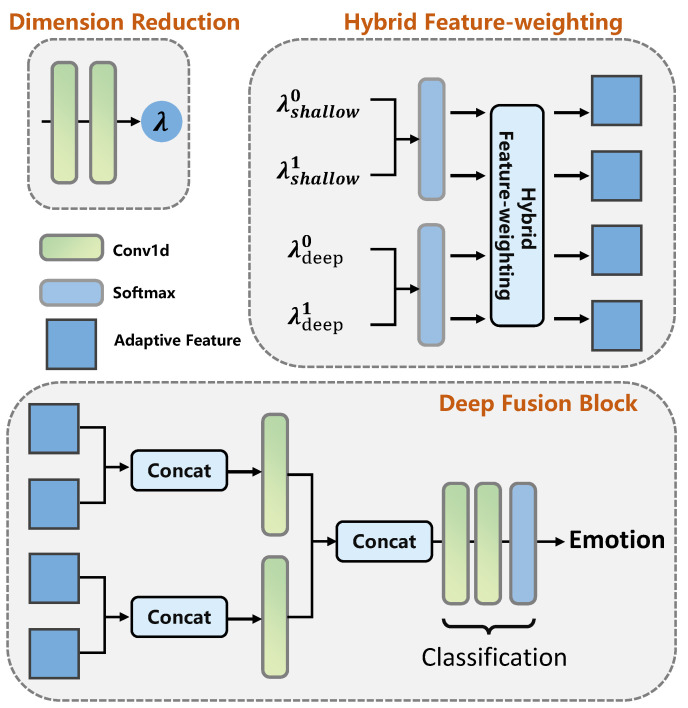
Adaptive-attention and deep fusion blocks for inferring optimal fusion weights and integrating the corresponding emotion adaptive features. The hybrid feature weighting is expressed as Equations (20) and (21).

However, the above process fails to filter ambiguous information, which is unfavorable for extracting salient emotion cues. It only utilizes the shallow features and their dimension-reduced weights without sufficiently considering the valuable complementary gains between face and context information. Therefore, in our work, supported by the informative complementary features from the I-R pair, we propose a novel AA-based fusion block, as shown in Figure 4, to alleviate this limitation and to further enrich the emotion information of the fused features by using the complementary gains between the face and context information. Different from the pipeline expressed in Equation (16) and Equation (17), we not only utilize the deep abstract features, corrected with complementary information between face and context features, to generate the adaptive features such as the items in the bracket of Equation (17), we also account for information compensation by introducing shallow feature maps of face and context, which ensures the model is free from overfitting to a certain extent and further improves the network’s robustness. To this end, the process of our proposed AA block can be expressed as in Equations (18)–(21). The details of the AA block are presented in Figure 7

(18) $$\lambda_{shallow}=\sigma \left(\prod \left(C_{1}\left(C_{1}\left({\tilde{X}}_{F}\right)\right),C_{1}\left(C_{1}\left({\tilde{X}}_{C}\right)\right)\right)\right),$$

(19) $$\lambda_{deep}=\sigma \left(\prod \left(C_{1}\left(C_{1}\left({\tilde{R}}_{F}^{CA}\right)\right),C_{1}\left(C_{1}\left({\tilde{R}}_{C}^{CA}\right)\right)\right)\right),$$

(20) $$f_{AA_{shallow}}^{i}=\lambda_{deep}\left[i\right]⊙{\tilde{X}}_{t},\;i=\left\{0,1\right\},t=\left\{C,F\right\},$$

(21) $$f_{AA_{deep}}^{i}=\lambda_{shallow}\left[i\right]⊙{\tilde{R}}_{t},\;i=\left\{0,1\right\},t=\left\{C,F\right\},$$

where $\lambda_{shallow}=\left[\left[\lambda_{shallow}^{F},\;\lambda_{shallow}^{C}\right]\right]$ and $\lambda_{deep}=\left[\left[\lambda_{deep}^{F},\;\lambda_{deep}^{C}\right]\right]$ are the optimal fusion weights. ${\tilde{X}}_{F}$, ${\tilde{X}}_{C}$, ${\tilde{R}}_{F}^{CA}$ and ${\tilde{R}}_{C}^{CA}$ and $R_{C}^{CA}$ are the respective feature vectors generated by GAP layers with ${\bar{X}}_{F}$, ${\hat{X}}_{C}$, $R_{F}^{CA}$ and $R_{C}^{CA}$ as inputs. $f_{AA_{shallow}}^{i}$ and $f_{AA_{deep}}^{i}$ are the four adaptive features generated by the AA block. For given (i=0,t=C) and (i=1,t=F), we can obtain the adaptive feature $f_{AA_{shallow}}^{0}$ and $f_{AA_{shallow}}^{1}$ using Equation (20). Repeating this process, we can obtain the rest of the adaptive features using Equation (21).

To combine the adaptive features generated with the AA block for final emotion classification, i.e., $f_{AA_{shallow}}^{0}$, $f_{AA_{shallow}}^{1}$, $f_{AA_{deep}}^{0}$ and $f_{AA_{deep}}^{1}$, a DF block is defined with hierarchical concatenation and classification parts (as presented in Figure 7), which can be expressed as Equations (22)–(26).

(22) $$f_{1}=Dropout\left(\delta \left(C_{1}\left(\prod \left(f_{AA_{shallow}}^{0},f_{AA_{deep}}^{0}\right)\right)\right)\right),$$

(23) $$f_{2}=Dropout\left(\delta \left(C_{1}\left(\prod \left(f_{AA_{shallow}}^{1},f_{AA_{deep}}^{1}\right)\right)\right)\right),$$

(24) $$X_{fusion}=\prod \left(f_{1},f_{2}\right),$$

(25) $$x_{cls}=\sigma \left(C_{1}\left(Dropout\left(\delta \left(C_{1}\left(X_{fusion}\right)\right)\right)\right)\right),$$

(26) $$p=argmax(x_{cls}),$$

where $X_{fusion}$ is the final fused feature for classification. $x_{cls}$ is the final output of network for classification, and *p* is the predicted label.

From the above pipeline of feature fusion, it can be seen that the process illustrated by Equations (16) and (17) can be treated as a particular case of the one by Equations (18)–(21), in which the inter-feature interaction performed by the CA block is neglected. Furthermore, as described above, the ER and AA blocks are successively introduced and developed to sufficiently utilize the complementary information obtained via the CA block and conduct the multi-feature fusion. Thus, with the removal of the CA block, the whole architecture of our proposed framework will shift to be consistent with the baseline method, as shown in Section 4.4.

### 3.4. Model Training Strategy

In the process of model training, the training set samples are fed into the proposed framework in the form of ${\left\{\left(X_{F}^{i},X_{C}^{i}\right),y_{i}\right\}}_{i=1}^{Ne}$, where $\left(X_{F}^{i},X_{C}^{i}\right)$ denotes the input pair corresponding to the facial region image and context image in the training set, and $y_{i}$ is the ground-truth label corresponding to the input pair. Ne is the number of training samples. To ease the problem of overfitting, the cross-entropy loss function with a flooding level [58] is utilized for parameter optimization, which can be expressed as:

(27) $$L_{C}=-\frac{1}{Ne}\sum_{i=1}^{Ne}\sum_{j=1}^{K}\log\mathrm{Pr}\left(y_{i}=j|\left(X_{F}^{i},X_{C}^{i}\right);\Theta \right)+\alpha ,$$

where α is the hyper-parameter of the flooding level. $\mathrm{Pr}\left(y_{i}=j|\left(X_{F}^{i},X_{C}^{i}\right);\Theta \right)$ is the probability that the input pair belongs to the *j*-th class, and *K* denotes the total number of emotion classes. Since the above function is continuously differentiable, we utilize the SGD optimizer with Nesterov momentum to obtain the optimal parameters [59]. Algorithm 1 details the overall model training process of CAHFW-Net.

**Algorithm 1:** CAHFW-Net.

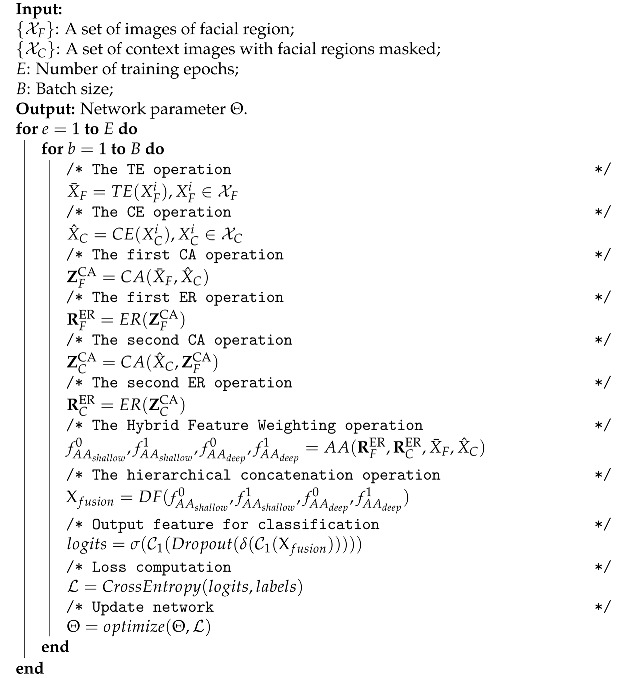



## 4. Experiments

To verify our idea and evaluate the effectiveness of the proposed CAHFW-Net, we conducted experiments on the publicly available context-aware emotion recognition dataset, namely the CAER-S dataset [19]. This section presents detailed information on the CAER-S dataset, followed by the implementation details of CAHFW-Net. We then compare the performance of our approach with some baseline algorithms. Finally, ablation studies are conducted to demonstrate the effects of different blocks.

### 4.1. Database and Evaluation Metrics

To overcome the lack of large-scale emotion recognition datasets, including spontaneous and unconstrained human faces and various context information in the wild, a static image dataset, called CAER-S, is extracted and created from 13,201 large-scale video clips containing around 1.1 M frames. It has about 70,000 images manually annotated with seven emotion categories, i.e., anger, disgust, fear, happy, neutral, sad, and surprise. For a fair comparison, we use the same split of 70%, 10%, and 20% for the training, validation, and testing sets as in Lee et al. [19].

The experimental results are reported with a widely used indicator, namely accuracy, to evaluate the prediction performance on discrete emotion categories in CAER tasks. Accuracy describes the deviation between the distribution of predicted and true categories, i.e., reporting the overall performance. The higher the accuracy value, the better the experiment performance. Equation (28) defines the calculation formulas for accuracy, where $T_{P}$, $F_{P}$, $T_{N}$ and $F_{P}$ indicate true_positive, false_positive, true_negative and false_negative, respectively.

(28) $$Accuracy=\frac{T_{P}}{T_{P}+F_{P}+T_{N}+F_{N}},$$

### 4.2. Experiment Setup and Implementation Details

#### 4.2.1. Implementation Details

The implementation of our proposed framework, which is deployed and accelerated on an AMAX GPU server with 4 NVIDIA GPUs (GeForce RTX 2080 Ti), is based on the PyTorch library [60]. We use the SGD optimizer with parameter values (0.9, True) for (momentum and nesterov). We trained CAHFW-Net from scratch with a learning rate initialized as 0.01 and dropped by a cosine annealing mechanism. As the primary sizes of face regions cropped by the Dlib CNN face detector and the context images are 96×96 and 400×712, for meeting the size requirement of the CA block, we resize $I_{F}$ and $I_{C}$ to 224×224. To reduce the effects of overfitting and avoid the phenomenon of validation loss and accuracy divergence, we employ a hyperparameter called flooding level [58] to restrict the bottom of the calculation of the training loss function. At the same time, dropout schemes with a default ratio of 0.5 are introduced in the DF block.

It is evident from the description in Section 3 that the parameters in the experiment are included in the construction of the DBE network, HAE network, and the deep fusion of adaptive features. Hence, in the following, we elaborate on the parameter settings in these three processes.

#### 4.2.2. The Network Structure of DBE and HAE networks

The overall architecture of our proposed DBE and HAE networks is illustrated in Figure 4. In this network, the TE and CE blocks in the DBE network are used to generate shallow representations of the face and context channels. The feature extraction part of the TE and CE blocks includes five Conv2D layers, which are set as (filters = 32, kernel_size = 3 × 3), (filters = 64, kernel_size = 3 × 3), (filters = 128, kernel_size = 3 × 3), (filters = 256, kernel_size = 3 × 3) and (filters = 256, kernel_size = 3 × 3). The context attention inference module of the CE block is constructed with three Conv2D layers with the setting of (filters = 256, kernel_size = 3 × 3), (filters = 128, kernel_size = 3 × 3) and (filters = 1, kernel_size = 3 × 3). The above 2D convolution layers have the same values (1, 1) for (stride, padding).

A single interactive operation between the face and context features in the HAE network is constructed with a CA block and an ER block. For the CA block, Figure 6 illustrates the corresponding structure. The four Conv2D layers at the transformation and output stages have the same setting, namely 256 filters with a size of 3 × 3 for one kernel, stride, and padding sizes of 1, 1. For the GIE of the ER block, as expressed in Equation (13), the Conv1D layer has 256 filters with a kernel_size of 1. The AA block executes the generation of adaptive features (as presented in Figure 4 and Figure 7), which has eight Conv1d layers with setting of (filters = 128, kernel_size = 1) and (filters = 1, kernel_size = 1) for each pair.

#### 4.2.3. Deep Fusion of Adaptive Features

The DF block for integrating the adaptive features generated by the AA block is constructed with the following two parts: hierarchical concatenation and classification. The hierarchical concatenation includes two linear layers with the same weight setting of $W\in R^{128\times 512}$ and concatenation at the last dimension of tensors. The classification part is built with two Conv1D layers, which are set as (filters = 128, kernel_size = 1) and (filters = 7, kernel_size = 1). In the above layers in the DBE network, HAE network, and deep fusion block, ReLU is used as the activation function. The value of the flooding level in the training loss function is 0.05.

### 4.3. Comparisons to Baseline Methods

**Figure 8:**
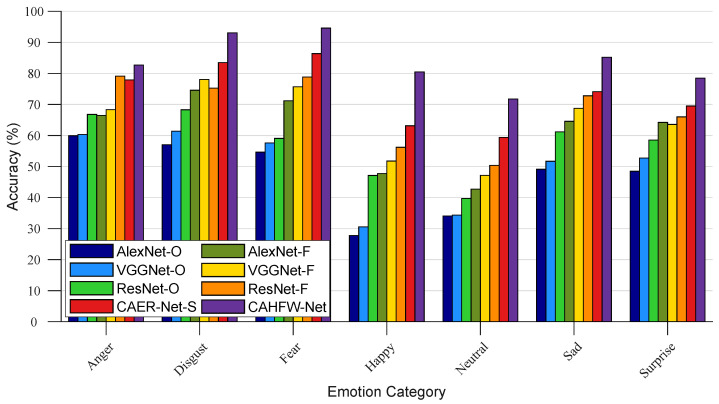
Comparison of emotion prediction performance with baseline methods on each category in the CAER-S benchmark. O and F denote off-the-shelf and fine-tuned versions, respectively.

In this section, we compare our proposed method with some baseline works, i.e., AlexNet-based [52], VGGNet-based [61], ResNet-based [53] and CAER-Net-S [19] methods, on the test set of the CAER-S dataset to illustrate the effectiveness of CAHFW-Net. The corresponding experimental results are presented in Table 2 and Figure 8. In the table, off-the-shelf and fine-tuned indicate the models pre-trained on the ImageNet dataset and fine-tuned on the CAER-S dataset, respectively. The CAER-Net-S and our CAHFW-Net are trained from scratch on the CAER-S dataset. From these comparisons, both from the macro (i.e., average accuracy) and micro (i.e., accuracy on each emotion category) view, we can determine that our proposed approach achieves a better experimental performance than these baseline methods.

From the macro perspective (i.e., average accuracy), it can be observed that the emotion recognition performance of the models fine-tuned on the CAER-S dataset is generally better than that of the off-the-shelf models only pre-trained on large-scale image datasets, where the ResNet-based network, with its more robust ability to resist network degradation and more vital feature abstractions, performs better overall than the other methods under the same training conditions. Furthermore, compared to the ResNet-based networks, the average prediction accuracy of CAER-Net-S increases by around 5.05% by benefiting from mining visual emotion cues in context, as in the work of [19]. From the table, we can observe that our proposed CAHFW-Net significantly outperforms the baseline methods with an accuracy of 83.76%. This is because the HAE network in the deep encoding stage jointly extracts and exploits emotion-related information from the perspective of inter-feature interaction and hybrid feature fusion.

Additionally, from the micro perspective (i.e., accuracy for each emotion category), we can see that the prediction accuracy of ResNet-F on the category Disgust is inferior to VGGNet-F. Similarly, the prediction accuracy of CAER-Net-S on the category Anger is lower than ResNet-F. These results partly reveal the limitations of the baseline methods in extracting discriminative emotion cues for a specific emotion category. In contrast, our proposed CAHFW-Net consistently performs favorably against baseline networks on each category in the CAER-S benchmark, as shown in Table 3. Specifically, compared to CAER-Net-S, the performance of CAHFW-Net on each emotion category significantly increases by approximately 4–17%. The accuracy of Happy and Neutral increase by around 17.35% and 12.35%, respectively, which further demonstrates that our proposed approach has a greater sensitivity and discrimination ability to different emotion categories using complementary information between different views of features via the CA and ER blocks.

### 4.4. Ablation Analysis

**Figure 9:**
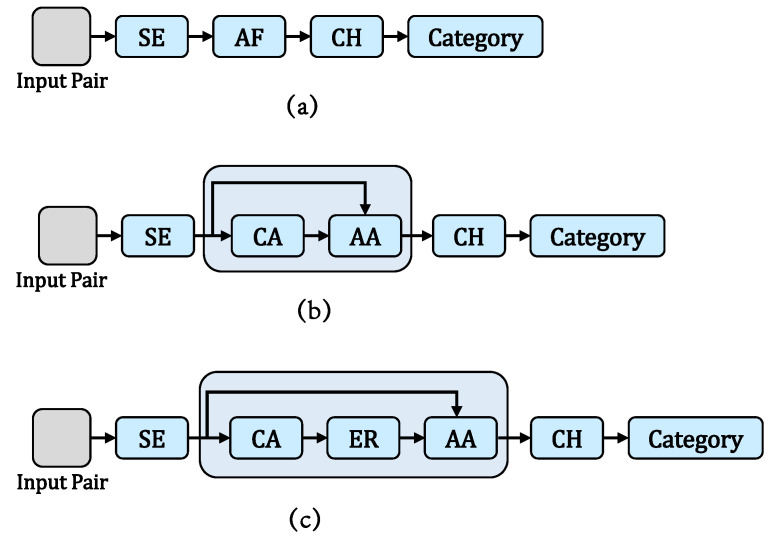
Different network architectures are used to investigate the role of proposed CA, AA, and introduced ER blocks for emotional state prediction. (**a**,**b**) denote the network without the CA, ER and AA blocks and the CAHFW-Net without the ER block, respectively, while (**c**) denotes the complete CAHFW-Net.

As previously discussed, the proposed model employs the complementary information between face and context features via the HAE network(including the CA, ER, and AA blocks) to predict the emotional state using a deep fusion strategy after obtaining the adaptive features. Hence, in this section, we conduct some necessary ablation experiments to demonstrate the role played by the three blocks (CA, AA, and ER) on the CAER-S dataset. The ablated architectures of networks are presented in Figure 9. In all three networks, the Input Pair indicates the image pair of face-context inputted into the SE block for shallow feature encoding. Note that the output is the discrete category of emotion and the loss functions for the three networks are all cross-entropy, as expressed in Equation (27). The SE refers to the shallow encoding block constructed with the TE and CE blocks. CA, ER, AA, and AF stand for cross-attention, element recalibration, adaptive-attention, and adaptive fusion (as expressed in Equations (16) and (17)), respectively. CH refers to the classification head (i.e., the classification part of the DF block).

#### 4.4.1. The Joint Role of the Cross-Attention and the Adaptive-Attention Blocks

To carefully explore the complementary benefit of the emotion inference of individuals between facial and contextual information, we construct a CA block to optimize the understanding of information strongly correlated with emotion in the facial and contextual regions from the perspective of feature interaction and complementary enhancement. From the description in Section 3.3, it is suggested that the AF block in the work of Lee et al. [19] is a particular case of our proposed framework, which neglects the effect of the complementary gains between face and context features. Furthermore, the AA block, which is used to integrate the adaptive features and is used for emotion inference, can be regarded as a twin block for the CA block, which is similar to the AF block. In other words, in the network (as shown in Figure 9b), the AA block is equivalent to the AF block when the CA block is removed. Thus, to jointly illustrate the effectiveness of the CA and AA blocks, we use the networks presented in Figure 9a,b to conduct the experiments. The corresponding results on the test set of the CAER-S dataset are presented in Table 4.

**Figure 10:**
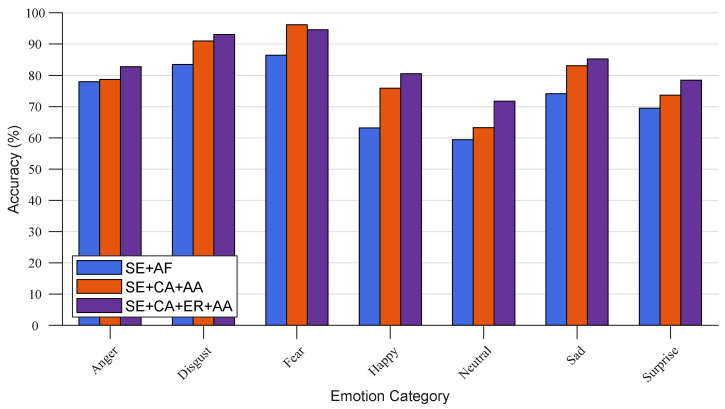
Comparison of emotion prediction performance among ablation networks as shown in Figure 9 on each emotion category in the CAER-S benchmark.

From Table 4, we can observe that the performance of networks SE + CA block + AA block (as shown in Figure 9b) is superior to that of SE blocks + AF block (as shown in Figure 9a). This result reveals that the CA block effectively captures the correlation and complementation between the features of the facial and contextual view. In contrast, the features used for inference are efficiently integrated by the AA block, which can boost the network’s capacity to infer the emotional state. Figure 10 presents the prediction accuracy of the aforementioned two networks on the seven emotion categories, which also verifies that compared to the network that uses a single-level simple concatenation for feature fusion only, a network with the CA and AA blocks, which perceives and utilizes the complementary gains between the two views of face and context, can significantly boost the emotion recognition performance.

#### 4.4.2. The Role of the Element Recalibration Blocks in the I-R Pair

The purpose of introducing the ER block in the I-R pair of the HAE network is to boost the representation of each branch by sufficiently embedding the global information using the aforementioned complementary information from the CA block. In other words, we highlight the feature regions related to the emotion cues and suppress the less valuable ones. Thus, comparative experiments with the network architectures shown in Figure 9b,c, are conducted to illustrate the effectiveness of the ER block. The experimental results are presented in Table 4.

**Figure 11:**
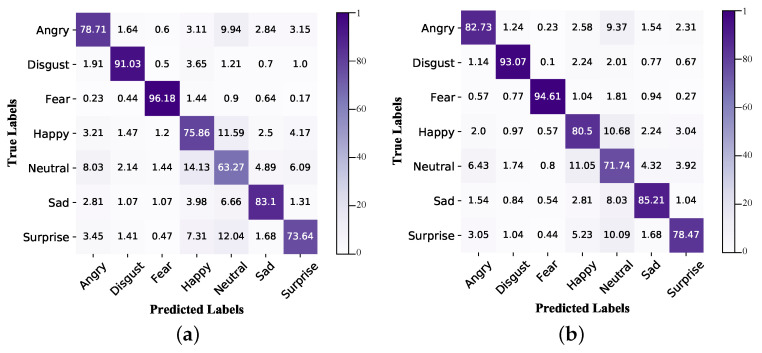
Confusion matrices of SE + CA + AA and SE + CA + ER + AA on the CAER benchmark. (**a**) SE + CA + AA; (**b**) SE + CA + ER + AA.

From Table 4, SE + CA + ER + AA (as shown in Figure 9c) achieves the best experimental performance and is better than SE + CA + AA in terms of prediction accuracy. The confusion matrices and prediction performance of the seven categories of SE + CA + AA and SE + CA + ER + AA are presented in Figure 10 and Figure 11. The aforementioned networks refer to CAHFW-Net without the ER block and CAHFW-Net, respectively. The reason for this result is that the ER block can examine all feature elements of tensors individually in each branch to pick out the salient ones and capture the subtle emotional cues. Furthermore, the better and more balanced performance of CAHFW-Net on each emotion category further verifies that the implying abundant complementary information, which is beneficial for the model to promote the understanding of emotion cues in the face and context features, is efficiently utilized by the ER block to generate deep abstract features.

## 5. Conclusions

To exploit discriminative complementary information between facial and contextual features in improving the performance of emotion predictions, in this paper, we propose a novel framework called CAHFW-Net for context-aware emotion recognition. Specifically, the I-R pair module is proposed in CAHFW-Net, in which the CA block focuses on seeking inter-feature complementary information by mining the correlation and heterogeneity between the face and context features. Following the CA block, the ER block is introduced to boost the network’s emotion representation ability by recalibrating the feature map of each channel using global information. Furthermore, to efficiently integrate the features, the AA block, with its core operation of hybrid feature weighting, is defined to obtain the optimal weighted features, which are further fused via a DF block in a hierarchical and dense manner for final emotion classification. The experiment results on the publicly available CAER-S emotion dataset verify not only the effectiveness of each block but also the superiority of our proposed method in the field of context-aware emotion recognition. In the future, we will try to extend our approach to more datasets, including videos, and utilize emotional representations in the dimensional space [62] (e.g., Valance, Arousal, and Dominance) to evaluate the emotional states from multiple perspectives. Additionally, we will integrate the proposed model with its potential applications, such as the analysis of tourist reviews with video clips, or the estimation of job stress levels with visual emotional evidence, or the assessment of mental health with visual media.

## Figures and Tables

**Table 1:** Summary of the mathematical notations used in this paper.

Symbol	Notation
*N*	The number of images
$I_{i}$	The *i*-th image
$I_{F}^{i}$	The *i*-th face image
$I_{C}^{i}$	The *i*-th context image with masked facial region
$bbox_{face}$	The bounding box of a facial region
$F_{F}^{i}\left(.\right)$	The *i*-th CNN block of TE block
$F_{C}^{i}\left(.\right)$	The *i*-th CNN block of front part of CE block
${\bar{X}}_{F}$	The output of TE block
${\bar{X}}_{C}$	The output of front part of CE block
$F_{AH}^{i}\left(.\right)$	The *i*-th CNN block of the attention-based highlight module
$C_{1}\left(.\right)$	The one-dimensional convolution layer
$C_{2}\left(.\right)$	The two-dimensional convolution layer
B(.)	The batch normalization layer
M(.)	The max pooling layer
δ(.)	The ReLU function
σ(.)	The softmax function
*A*	The attention map of attention-based highlight module
${\hat{X}}_{C}$	The output of CE block
$Y_{F}^{\mathrm{CA}}$	The output of first cross-attention operation
$Z_{F}^{\mathrm{CA}}$	The output of first CA block
$Q_{F},Q_{C}$	The query of ${\bar{X}}_{F}$ and ${\hat{X}}_{C}$
$K_{C},K_{Z_{F}}$	The key of ${\hat{X}}_{C}$ and $Z_{F}^{\mathrm{CA}}$
$V_{F},V_{C}$	The value of ${\bar{X}}_{F}$ and ${\hat{X}}_{C}$
$F_{flatten}\left(.\right)$	The flatten operation
$F_{Q,K,V}$	The function to obtain query, key and value
$\bar{Z_{F}}$	The tensor flattened from $Z_{F}^{\mathrm{CA}}$ in the last dimension
$W_{F}^{\mathrm{TM}}$	The transformation matrix in GIE of ER block
$G_{F}$	The global information of feature map $Z_{F}^{\mathrm{CA}}$
$R_{F}^{\mathrm{ER}}$	The output of first ER block
λ	The fusion weight list of adaptive fusion network
${\tilde{X}}_{F}$	The tensor after operating global average pooling on ${\bar{X}}_{F}$
${\tilde{X}}_{C}$	The tensor after operating global average pooling on ${\hat{X}}_{C}$
$X_{fusion}$	The fused feature for classification
∏(x)	The concatenation operator
${\tilde{R}}_{F}^{CA}$	The tensor after operating global average pooling on $R_{F}^{CA}$
${\tilde{R}}_{C}^{CA}$	The tensor after operating global average pooling on $R_{C}^{CA}$
$\lambda_{shallow}$	The fusion weight list generated from ${\tilde{X}}_{F}$ and ${\tilde{X}}_{C}$
$\lambda_{deep}$	The fusion weight list generated from ${\tilde{R}}_{F}^{CA}$ and ${\tilde{R}}_{C}^{CA}$
$f_{AA_{shallow}}^{i},f_{AA_{deep}}^{i}$	The emotion adaptive features
$f_{1},f_{2}$	The intermediate fused feature in deep fusion block
$x_{cls}$	The output feature of network
$p_{i}$	The *i*-th predicted label
$y_{i}$	The *i*-th true label
$T_{P}$, $F_{P}$, $T_{N}$, $F_{P}$	True_positive, False_positive, True_negative, False_negative

**Table 2:** Quantitative evaluation of CAHFW-Net in comparison to baseline methods on the test set of the CAER-S benchmark.

Methods	Acc. (%)
Off-the-shelf AlexNet [52]	47.36
Off-the-shelf VGGNet [61]	49.89
Off-the-shelf ResNet [53]	57.33
Fine-tuned AlexNet [52]	61.73
Fine-tuned VGGNet [61]	64.85
Fine-tuned ResNet [53]	68.46
CAER-Net-S [19]	73.51
**CAHFW-Net (ours)**	83.76

**Table 3:** Accuracy (%) of CAHFW-Net and baseline methods on each emotion category in the CAER-S benchmark.

Emotions	AlexNet		VGGNet		ResNet		CAER-Net-S	CAHFW-Net
	Off-the-Shelf	Fine-Tuned	Off-the-Shelf	Fine-Tuned	Off-the-Shelf	Fine-Tuned	from Scratch	from Scratch
Anger	59.92	66.49	60.34	68.34	66.80	79.14	77.93	82.73
Disgust	57.01	74.59	61.40	78.08	68.29	75.28	83.49	93.07
Fear	54.62	71.20	57.59	75.70	59.07	78.83	86.40	94.61
Happy	27.79	47.74	30.58	51.77	47.11	56.21	63.15	80.50
Neutral	34.08	42.71	34.34	47.16	39.74	50.39	59.39	71.74
Sad	49.12	64.58	51.71	68.76	61.19	72.79	74.11	85.21
Surprise	48.48	64.21	52.72	63.57	58.54	66.01	69.51	78.47

**Table 4:** Performance of emotion prediction on CAER-S test set using different networks. The networks of SE block + AF block and SE block + CA block + AA block are shown in Figure 9. The network of SE block + CA block + ER block + AA block is shown in Figure 4.

Network Structures	Acc. (%)
SE blocks + AF block	73.51
SE blocks + CA block + AA block	80.26
**SE blocks + CA block + ER block + AA block**	83.76

## Data Availability

Not applicable.
